# Radiosensitizing effect of diosmetin on radioresistant lung cancer cells via Akt signaling pathway

**DOI:** 10.1371/journal.pone.0175977

**Published:** 2017-04-17

**Authors:** Zhijie Xu, Yuanliang Yan, Lingfang Xiao, Shuang Dai, Shuangshuang Zeng, Long Qian, Lin Wang, Xue Yang, Yi Xiao, Zhicheng Gong

**Affiliations:** 1 Department of Pathology, Xiangya Hospital, Central South University, Changsha, China; 2 Department of Pathology, School of Basic Medicine, Central South University, Changsha, China; 3 Department of Pharmacy, Xiangya Hospital, Central South University, Changsha, China; 4 Institute of Hospital Pharmacy, Central South University, Changsha, China; University of South Alabama Mitchell Cancer Institute, UNITED STATES

## Abstract

Radiotherapy is a powerful tool in the treatment of cancer that has the advantage of preserving normal tissues. However, tumor radioresistance currently remains a major impediment to effective RT. Thus, exploring effective radiation sensitizers is urgently needed. In this study, we have shown that diosmetin, the aglycone of the lavonoid glycoside from olive leaves, citrus fruits and some medicinal herbs, has a promising effect on radiotherapy sensitization. In our results, DIO could induce G1 phase arrest and thus enhance the radiosensitivity of radioresistant A549/IR lung cancer cells. Furthermore, DIO also restrains the IR-induced DNA damage repair by inhibiting the activated Akt signaling pathway. The combination of Akt inhibition (DIO, LY294002 or MK-2206) and radiation potently blocked A549/IR cancer cell proliferation. In summary, these observations suggest that the natural compound DIO could act as a potential drug for the treatment of radioresistant lung cancer cells.

## Introduction

Radiotherapy is a promising treatment strategy for early-stage or advanced-stage lung cancer patients. Despite being treated with RT, some patients with higher operative and surgical risks often experience recurrence and metastatic diseases [1, 2]. One main factor for these unsatisfactory therapeutic outcomes following RT is because of radioresistant profiles in a subpopulation of cell clones within the neoplasm. Therefore, radioresistance is currently considered to be a major challenge for the therapeutic efficacy in lung cancer [3, 4]. Strategies for improving the response ratio of RT are warranted to minimize the radioresistance influence on cancer cells.

The protein kinase B (PKB/Akt) signaling pathway is frequently hyperactivated during tumorigenesis and has been proven to be a candidate target for cancer therapy [5]. As an important intracellular signaling molecule, Akt is crucial for cell survival and growth, particularly during cancer progression and radioresistance [6]. Thus, attenuating Akt activation by several pharmacologic assays could demonstrate excellent anticancer effects. Li et al. demonstrated that inhibition of Akt by inhibitor MK-2206 and platycodin D could potentiate proliferative inhibition and apoptotic induction in lung cancer cells [7]. Zhang et al. found that fisetin, a dietary phytochemical, overcomes therapy resistance of lung cancer cells through inhibition of Akt pathways [8]. Specific inhibition of Akt with triciribine significantly facilitates the damaging effects of radiation in H460 lung cancer cells [9]. In addition, recent studies have suggested that Akt inhibition results in a concomitant decrease in the abundance of key DNA repair genes, which are responsible for DNA-damage repair upon radiation stress. Blocking Akt signaling by small interfering RNA (siRNA) provokes DNA damage and induces the cell-cycle arrest in acute lymphoblastic leukemia [10]. Similarly, bevacizumab could cause DNA double-strand breaks (DSBs) by suppressing Akt activation, further sensitizing the lung cancer cells to RT [11].

Diosmetin [12], a flavone found in legumes and in olive leaves, shows attractive cytotoxic activity on human cancer cells. The result from Androutsopoulos et al. suggested that DIO induces anticancer activity in MCF7 breast cancer cells by causing cytochrome P450 bioactivation [13]. Meanwhile, Zhan et al. found that DIO could induce G1/S arrest and cell apoptosis in human lung cancer A549 cells [14]. However, no reports have been published to test agent DIO as a radiosensitizer to improve radiosensitivity so far. The underlying mechanisms of DIO in combination with RT in the treatment of lung cancer cells remain to be fully elucidated.

Here, we investigated (1) whether cell cycle distribution, cell cycle checkpoint proteins, and cell survival are affected by DIO administration and (2) whether the Akt signaling pathway and DNA-damage response are associated with the radiosensitivity of lung cancer cells after treatment with a combination of DIO and RT.

## Materials and methods

### Cell lines and reagents

The lung cancer radioresistant cell line, A549/IR, in our study was a gift from the Winship Cancer Institute of Emory University [15]. In brief, the A549/IR cell line was established by exposing A549 cells in exponential growth phase to a repeated IR dose of 4 Gy each. An interval of 3 to 8 weeks between each IR dose allowed the surviving cells to regenerate. The whole process of IR and culture lasted for approximately 1 year, with a total IR dose of 80 Gy. We refer to the surviving cell line as A549/IR. Human lung cancer cell lines A549 [ATCC^®^ CCL-185™] and A549/IR were cultured in DMEM/F12 medium (Gibco, USA) supplemented with 10% fetal bovine serum (HyClone, USA), 1% penicillin and streptomycin. All cell lines were grown in a humidified incubator at 37°C with 5% CO_2_.

DIO (S2380), LY294002 (S1105) and MK-2206 (S1078) were purchased from the Selleck Chemicals (USA). All agents were dissolved in dimethylsulfoxide. The final concentrations of DIO, LY294002 and MK2206 used for the stimulation of cells were 10 μM, 20 μM and 10 μM, respectively. Additionly, the radiation processing time we used in this manuscript is 1h, which is in accordance with the previous findings [16].

### Western blot

The antibodies for western blot were as follows: anti-β-actin (8432, Santa Cruz, 1:2000), anti-CyclinD1 (753, Santa Cruz, 1:1000), anti-ATM (23921, Santa Cruz, 1:1000), anti-phospho-ATM (47739, Santa Cruz, 1:100), anti-p53 (126, Santa Cruz, 1:1000), anti-phospho-p53 (Ser15, 101762, Santa Cruz, 1:1000), anti-CDK4 (12790, Cell Signaling Technology, 1:1000), anti-CDK6 (13331, Cell Signaling Technology, 1:1000), anti-CDC2 (9116, Cell Signaling Technology, 1:1000), anti-Akt (9272, Cell Signaling Technology, 1:1000), anti-phospho-Akt (ser473, 4060, Cell Signaling Technology), anti-phospho-DNA-PK (Thr2609, 18356, Abcam), anti-DNA-PK(32566, Abcam) and anti-γ-H2AX (05–636, Millipore, 1:1000). Protein expression levels were determined with western blot as previously described [17].

### MTS assay

Approximately 1×10^3^ cells were seeded into 96-well plates and then incubated for 0, 24, 48, or 72 h. MTS reagent was added to each well, and cell viability was evaluated in A549 and A549/IR cell lines using a spectrometer according to the instructions provided (Promega, USA).

### Clonogenic survival assay

Approximately 2×10^3^ cells were seeded into a 6-well plate and incubated for 24 h. The cells were then treated with a range of IR doses (0, 2, 4, 8 Gy) using a gamma irradiator. After approximately 15 days, cells were washed with PBS, fixed in methanol and stained with crystal violet. Survival curves were generated using Microsoft Excel (Microsoft Office system, USA)

### Flow cytometric analysis

A flow cytometry was performed to compare the difference in cell cycle distribution between A549 and A549/IR cells using a published method [18]. Briefly, cancer cells (approximately 1×10^6^) were seeded onto 6-well plates and cultured with the indicated DIO concentrations (0.5 mM) for 24 h or exposed to 6 Gy IR for 1 h. Treated and untreated cells were collected, washed with ice-cold PBS and fixed in 70% ethanol, and then stored at 4°C overnight. The fixed cells were washed with PBS again and stained with 0.1% RNase A and 50 μg/ml propidium iodide in the dark at 25°C for 30 min and assayed on FACSort (Becton-Dickinson), and the cell cycle parameters were determined using the CellQuest software program (Becton-Dickinson). A minimum of 1×10^4^ cells were counted for each sample.

### Confocal microscope analysis

A549 and A549/IR cell lines were seeded on Millicell EZ slides (Millipore) and subjected to the following treatments: untreated, 6 Gy for 1 h or DIO for 24 h. After that, the cells were used for confocal microscope assays following the published method [18].

### Statistical analysis

All data were repeated at least three times with similar results and are expressed as the mean ± SD form. Comparisons between two groups were performed using Student’s t test with SPSS 12.0. P values <0.05 or <0.001 were considered statistically significant.

## Results

### Anti-proliferation effect of DIO on the radioresistant lung cancer cells A549/IR

**Fig 1:**
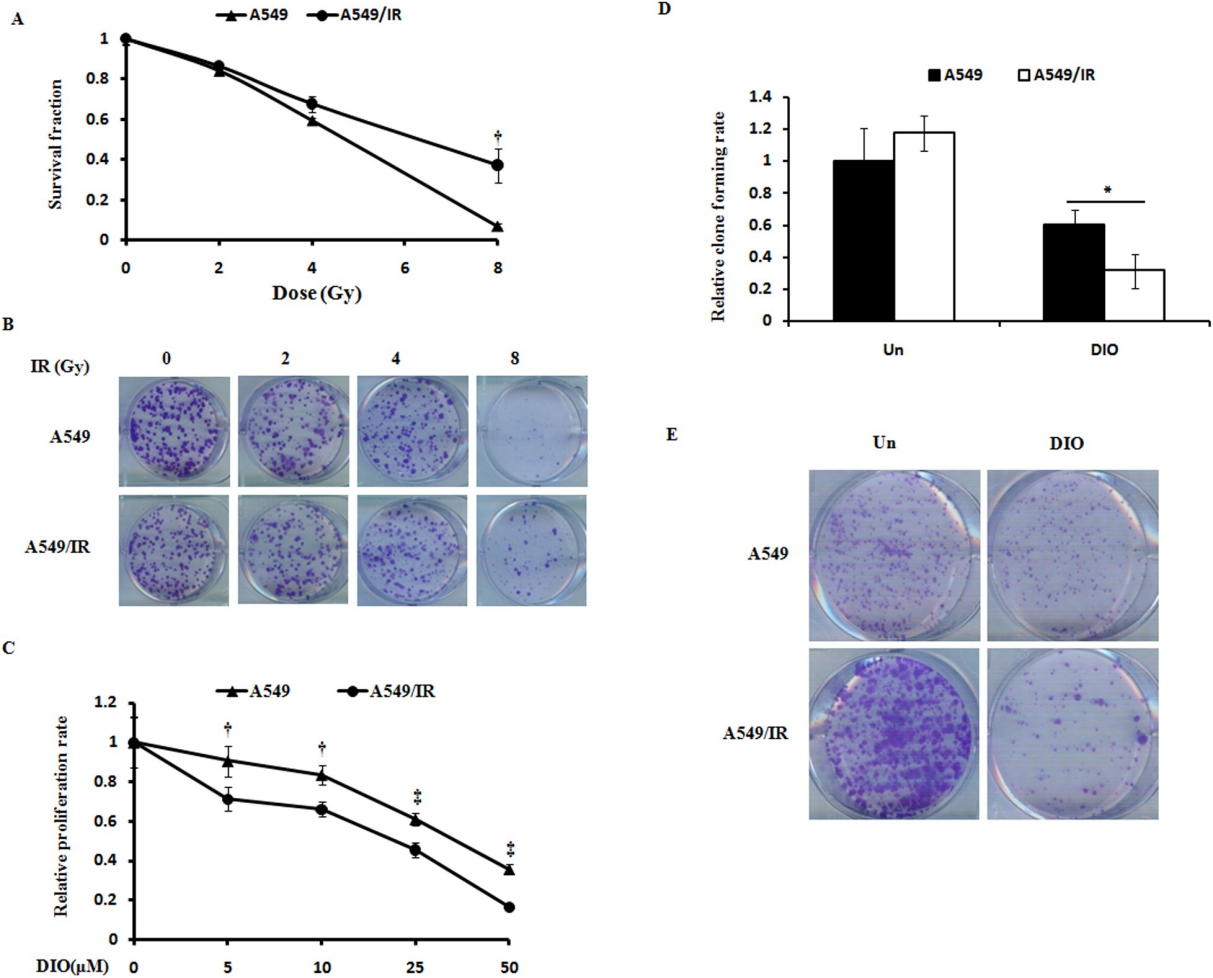
DIO suppresses the cell survival of radioresistant A549/IR cells. (A-B) One lung cancer radioresistant cell line, A549/IR, was established. The data represent the results of colony formation assays and survival fractions for A549 and A549/IR cell lines. (C) A549 and A549/IR cell lines were treated with different doses of DIO for 24, 48, and 72 h. Cell viability was evaluated using the MTS assay. (D-E) Colony formation assays show the effect of 10 μM DIO on A549/IR cells. The quantitative results shown of three independent experiments are the mean ± SD. *P<0.05. **P<0.01.

The A549/IR lung cancer radioresistant cell lines were established. To identify the radioresistant phenotypes, cells were irradiated with different doses of IR (0, 2, 4, 8 Gy). After approximately 12 days, we examined the cellular survival fractions using the clonogenic survival assay. Compared with the corresponding A549 cells, the SFs and colony formation of A549/IR were much higher after IR treatment (Fig 1A and 1B). These findings confirmed the radioresistant phenotype of A549/IR cell lines.

**Fig 2:**
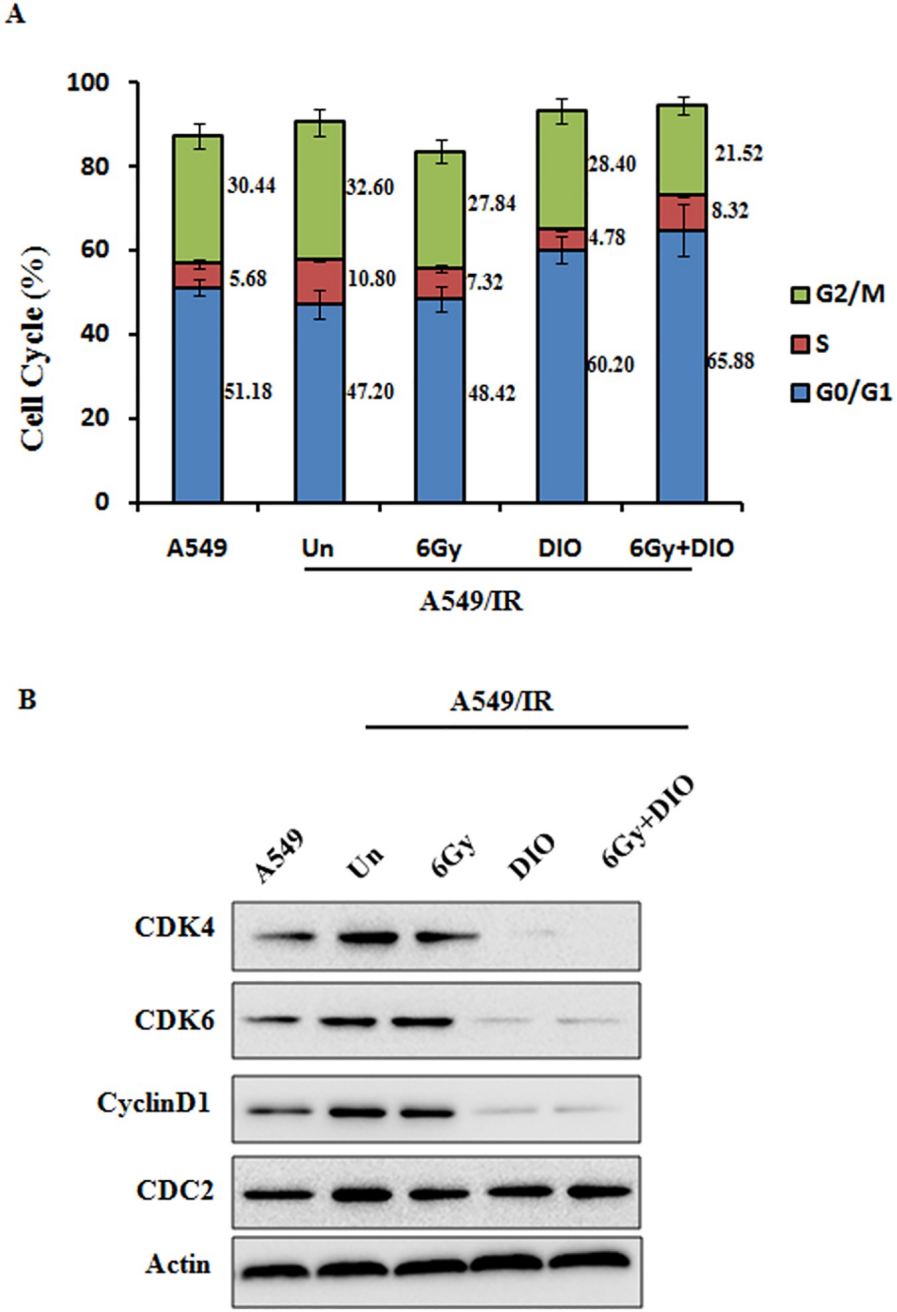
Effects of DIO on the cell cycle distribution in A549/IR cells. (A) A549/IR cells were treated with 10 μM DIO for 24 h, 6 Gy RT for 1 h, or a combination of DIO and RT, and then cell cycle distributions were analyzed with flow cytometry. The quantitative data of cell cycle distribution are shown. (B) Cell cycle-related proteins (CDK4/6, CyclinD1 and CDC2) were determined using western blot analysis. β-actin was used for the loading control.

To verify the effect of DIO on cell proliferation, we treated A549/IR and A549 cells with different doses of the compound DIO. DIO could effectively inhibit cell proliferation and clone formation in both A549 and A549/IR cell lines in a dose-dependent manner (P < 0.01). However, the inhibition ratio of DIO on A549/IR cells is more remarkable (Fig 1C–1E). Furthermore, we choose 10 μM as the best working concentration of DIO in the next experiments, which is in accordance with Zeng’s findings [19]. Since cell proliferation can be regulated by cell cycle progression [20], we next examined the effect of DIO on cell cycle distribution. DIO was administered to radioresistant A549/IR cells after overnight serum starvation, and cell cycle distribution was assessed by flow cytometry. DIO significantly increased the percentage of cells in G1 phase (60.20 ± 4.13) compared to untreated cells (47.20 ± 1.19). When DIO treatment was combined with 6 Gy, the percentage of cells in G1 phase could be increased to 65.88 ± 3.31. This increase was coupled with a significant decrease in the percentage of cells in G2/M phase (Fig 2A). It is well established that cyclin-dependent kinases (Cdks) and cyclin complexes are perceived as the engine that drives cell cycle progression [21]. To further clarify the G1 phase arrest phenotype due to DIO, we investigated the cell cycle-associated checkpoint. It was found that in A549/IR cells, DIO markedly decreased the expression of CDK4/6 and cyclin D1, the regulators required for G1/S transition [22], but had no obvious effect on the expression of CDC2, a critical regulator during G2 phase progression [23]. Moreover, the combination of DIO and radiation could further down regulate the G1 phase regulators, while radiation alone could not affect the cell cycle regulators in A549/IR, which further indicates the phenotype of radio-resistance in A549/IR cells (Fig 2B). In addition, we examined the effect of DIO on cell proliferation and cell cycle in the parent A549 cell lines. Expectedly, DIO and 6Gy IR could both significantly inhibit the cell proliferation rate and cell cycle-associated checkpoint makers CDK4/6 and cyclin D1, however the combination of DIO and IR have no obviously further cell-killing effects in A549 cell lines (S1A and S1B Fig). Taken together, these data suggest that DIO has a preferential anti-tumor effect on the radioresistant lung cancer cells.

**Fig 3:**
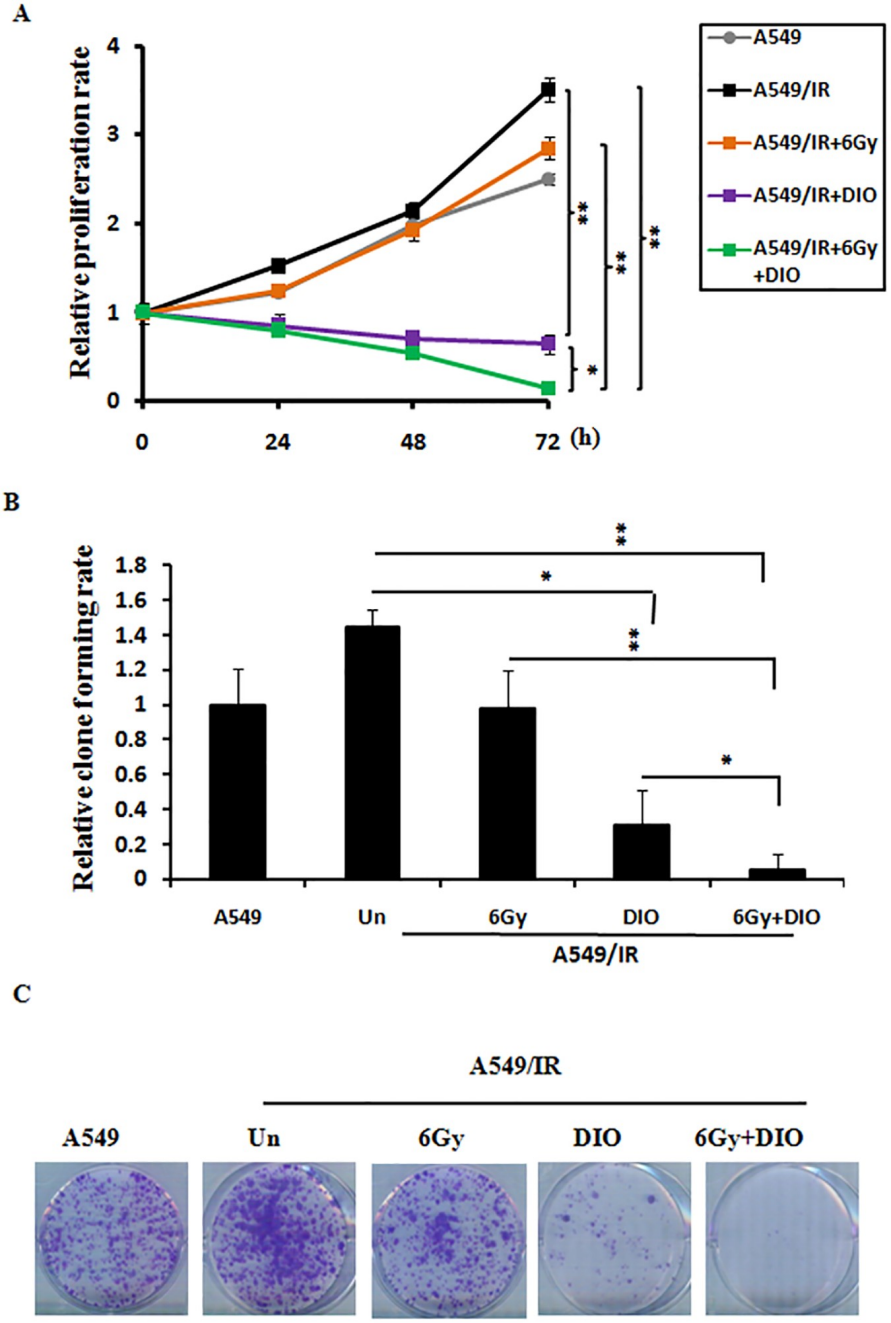
DIO enhances the radiosensitivity of A549/IR cells. (A) A549/IR cell lines were treated with indicated conditions for 24, 48, and 72 h. Cell viability was evaluated using the MTS assay. (B-C) A549/IR cells were treated by 10 μM DIO with or without 6 Gy IR. After 24 h, typical images of cell colony growth for the different treatments are shown. Values are the means ± SD of 3 replicates, *P < 0.05, **P < 0.01 compared with the control.

Next, cell viability and clonogenic survival assays were performed to provide further evidence for the role of DIO in the regulation of cell growth under irradiation. A549/IR cells were treated with DIO and subjected to X-ray irradiation. As shown in Fig 3A, when A549/IR cells were exposed to DIO, a significant decrease in cell proliferation was observed compared to that observed for untreated control cells. Moreover, a strong inhibition of growth occurred upon combination treatment of DIO and IR (P<0.01) (Fig 3B). The typical images for colony formation from different treatments are shown in Fig 3C. These results collectively show that DIO administration could result in significantly increased radiosensitivity in radioresistant lung cancer cells.

### DIO impaired IR-induced DNA damage repair in A549/IR cells

**Fig 4:**
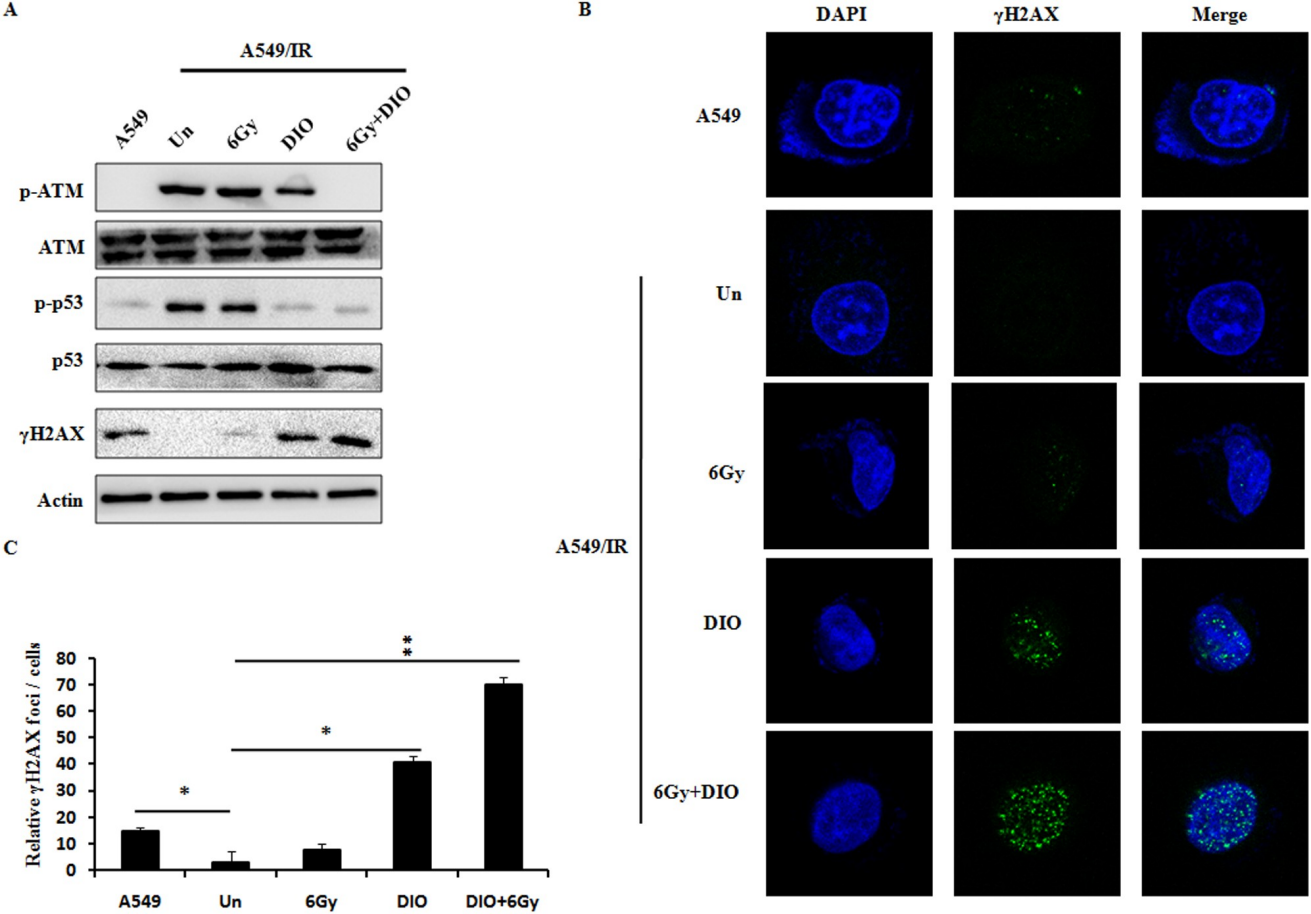
Effects of DIO on the DNA damage in A549/IR cells. (A) Reduced phosphorylation of ATM and p53, and increased γH2AX expression level upon DIO treatment with or without RT. Cell lysates were processed for the indicated proteins by immunoblotting. β-actin expression shows the equal loading. (B) γH2AX foci status was investigated by using a confocal analysis for different treatment. Representative images are shown. (C) Quantitative data of γH2AX foci are summarized. Bars represent the means ± SD of triplicate samples. *P < 0.05, **P < 0.01.

Previous reports show that IR-induced cell killing mainly depends on the radiation-induced DNA damage responses, and the ability of tumor cells to elicit a DNA damage response following radiation promotes the radioresistance and cell survival [24]. To determine the effects of DIO on modulating the activities of both the homologous recombination and non-homologous end joining (NHEJ) repair systems, two key DNA repair pathways [25], we first assessed the DNA damage signaling by western blot. As shown in Fig 4A and S2 Fig, after treatment with DIO alone, the γH2AX levels (DNA double-stand break maker) were significantly increased, while ataxia-telangiectasia mutated (ATM) phosphorylation, DNA-dependent protein kinase (DNA-PK) phosphorylation, p53 phosphorylation and RAD51 level (DNA-damage repair makers) were all reduced in A549/IR cells compared with untreated control cells. This finding from DNA-damage signal proteins indicated that DIO inhibited the DNA damage repair in A549/IR cells. To further confirm this result, γH2AX foci in A549/IR cells were well investigated next. As many proteins regulating the DNA damage response are known to form foci at the damaged DNA [26], we next performed immunofluorescence staining with antibodies against γH2AX. Foci formation of γH2AX was increased in the cells pretreated with DIO (Fig 4B). And the quantitative results and p-values are shown in Fig 4C. It is worth noting that the changes of DNA damage signaling, including the γH2AX foci, in A549/IR cells with a combination of DIO and IR were the most obvious. In addition, IR alone only induced slightly alterations of DNA-damage signaling, supporting the radioresistance phenotype of A549/IR cells. These results collectively indicated that the DIO-mediated DNA damage response is not only because of the direct induction but also because of the radiosensitization effect of DIO.

### Akt signaling is involved in DIO-mediated DNA-damage response in A549/IR cells

**Fig 5:**
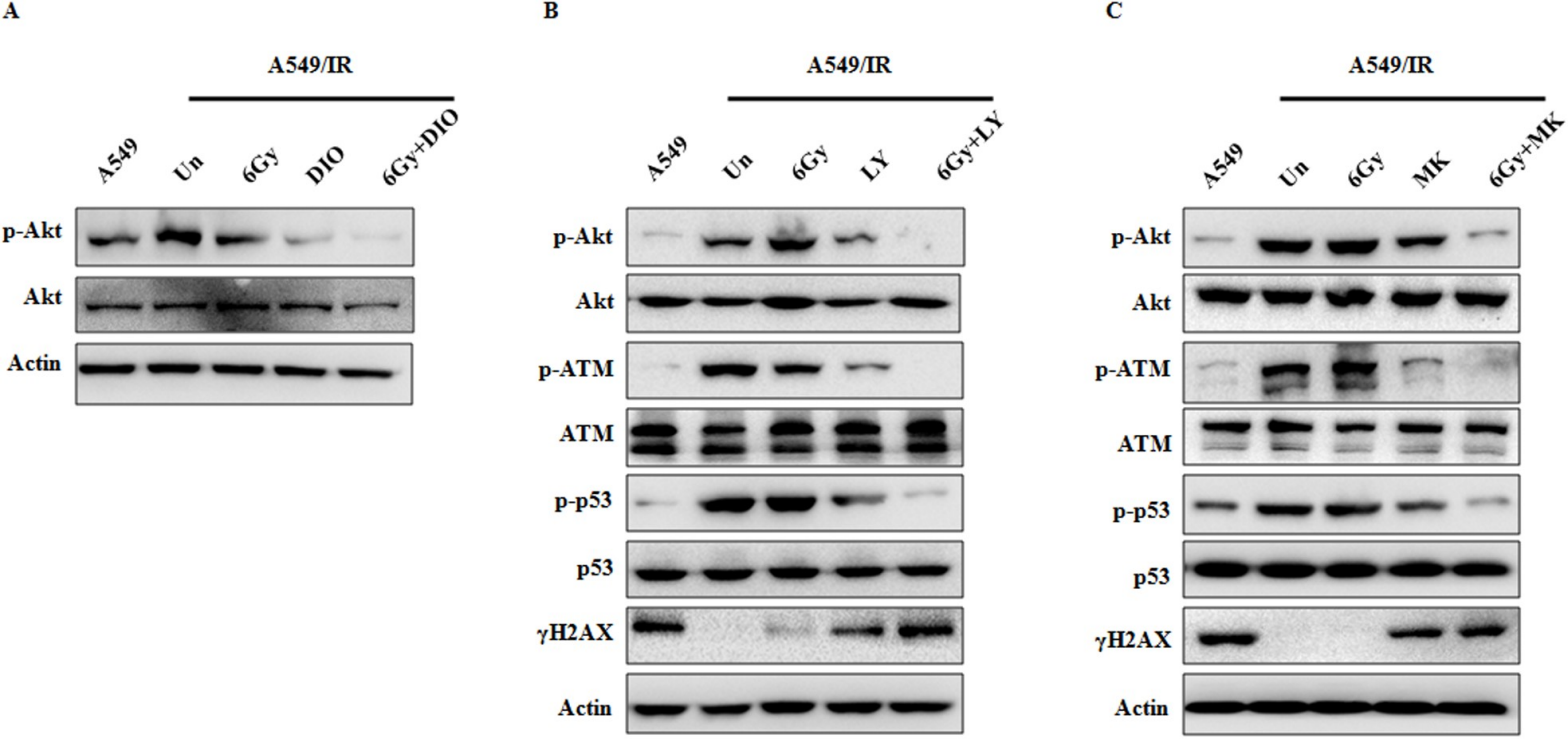
Radiosensitizing effect of DIO on A549/IR cells is dependent on Akt activation. A549/IR cells were treated by 10 μM DIO, 20 μM LY294002 and 10 μM MK-2206 for 24 h and irradiated at 6 Gy for 1 h. Then, the protein expression levels were analyzed by western blot with the indicated antibodies. β-actin used as an internal normalization control.

As recent studies have revealed that the Akt signaling pathway promotes radioresistance in human cancer cells through inducing the DNA damage repair pathways [27], we next examined the effects of DIO on Akt activation. The results show that in A549/IR cells, compared with the untreated group, decreased levels of Ser473 phosphorylated Akt can be seen upon DIO treatment, whereas no significant change was seen in the expression of total Akt. The combination of DIO and IR could further down-regulate the Akt Ser473 level (Fig 5A). To further investigate the role of Akt signaling in the DNA damage response, we inhibited Akt activity with two inhibitors in the A549/IR cells, LY294002 [28] and MK-2206 [29]. As expected, Akt inhibitors yielded similar results to DIO on the DNA damage response. Pharmacologic inhibition of Akt activation by LY294002 or MK-2206 resulted in a decrease of p-ATM and p-p53 level, and an increase of γH2AX level. In the meantime, after treatment with the combination of Akt inhibitors with IR, the changes of DNA damage modulators were the most significant (Fig 5B and 5C). Taken together, these data suggest that DIO inhibits DNA damage repair ability of radioresistant lung cells through interference with the Akt signaling pathway.

**Fig 6:**
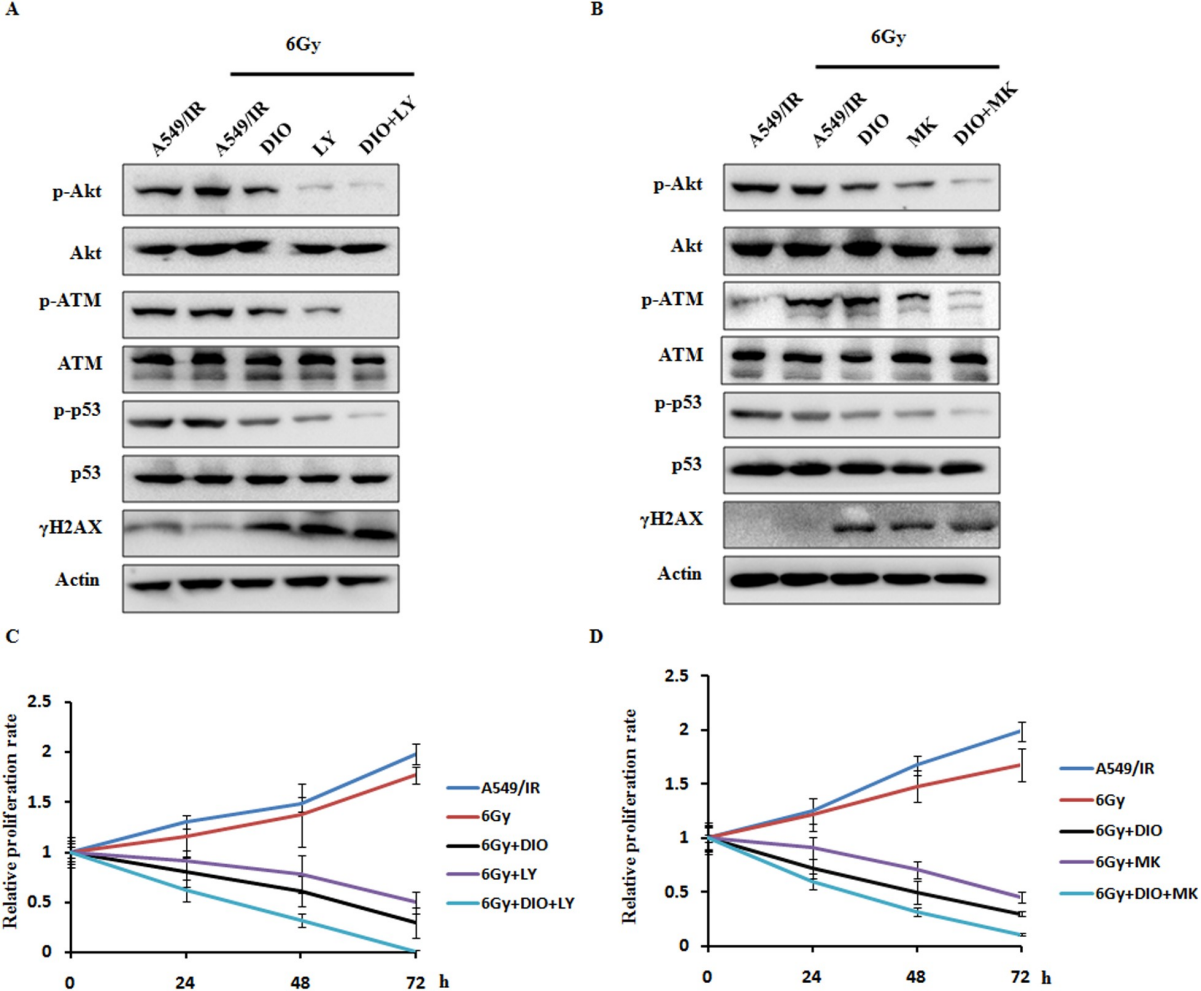
Inhibition of the Akt signaling pathway sensitizes lung cancer cells to radiation. (A-B) A549/IR cells were treated with a dual or single agent for 24 h and then treated with 6 Gy RT or directly treated with 6 Gy RT alone. Then, cell lysates were processed for the indicated proteins by western blot assay. (C-D) MTS assays showing the response of DIO and Akt inhibitors on the A549/IR cells to the radiation treatment. β-actin used as an internal normalization control. Similar results were obtained in *n* = 3 experiments.

To further identify if repressing Akt signaling could sensitize the cancer cells to radiotherapy, we examined the effect of Akt inhibitors LY294002 and MK-2206 on cell proliferation in A549/IR cells. Consistent with earlier reports after IR treatment, DIO and Akt inhibitors could both inhibit ATM and p53 phosphorylation expression and up-regulate γH2AX expression level. In addition, the most prominent changes in DNA damage response modulators could be clearly noted upon combination treatment of DIO and Akt inhibitors (Fig 6A and 6B). Additionally, to evaluate the role of Akt activity inhibition in the radiosensitivity of A549/IR cell lines, cell proliferation rates were measured by MTS cell proliferation colorimetric assay. The results showed that DIO and Akt inhibitors (LY294002 and MK-2206) could significantly enhance the anti-tumor effect of radiation. Meanwhile, a combination of DIO and LY294002 or MK-2206 could further enhance the radiosensitivity of A549/IR cells (Fig 6C and 6D). These findings demonstrated that the combination of Akt inhibitors with radiotherapy is a promising modality for the treatment of lung cancer cells to overcome radioresistance.

## Discussion and conclusion

In the current study, using the radioresistant A549/IR model and cancer cell biology techniques, we present novel insight into the mechanisms of compound DIO as a promising radiation sensitizer in lung cancers. In the first step, we demonstrate the association of DIO administration with cell cycle distribution and the DNA repair pathway in A549/IR cells. Then, studies on molecular mechanism revealed that DIO could enhance the radiosensitivity of lung cancer cells via inhibiting the Akt signaling pathway.

It is known that cell cycle changes impact the curative effect of radiotherapy in human cancer cells. According to the report by Marampon et al., down-regulation of Cyclin D1, a G1/S checkpoint regulator, impairs DSB repair and thus promotes the radiosensitivity of prostate cancer cells [12]. The antidiabetic drug metformin potently triggers G1 cell cycle phase arrest, and further enhances radiosensitivity in pancreatic cancer cells [30]. Moreover, Kriegs et al. found that epidermal growth factor receptor (EGFR) inhibition could induce cellular radiosensitization by enhancing the radiation-induced permanent G1 arrest in lung cancer cells A549 [31]. Additionly, the combination of DIO and IR slightly enhance the cell-killing effects in parent A549 cells, whereas DIO significantly promotes the sensibilization of radiotherapy in A549/IR cell lines. Thus, in line with the previous reports, our current data in cell cycle analysis show that DIO could effectively induce the G1 phase arrest, and finally sensitize A549/IR cells to radiation.

DNA damage has been proven to be the principal cytotoxic lesion of chemotherapy or ionizing radiation [32]. Even though a basal level of γH2AX could be seen in the pair cells, the percentage of γH2AX-positive cells was very low in non-irradiated cancer cells. We found that the key proteins, including reduced ATM, p53, RAD51 and DNA-PKcs, and increased γH2AX in A549/IR cells treated with DIO and RT, which implies that the DNA repair pathway plays an important role in the regulation of DIO-mediated radiotherapy sensitization in lung cancer cells. Meanwhile, 6Gy IR alone have no obvious effect on the DNA-damage associated factors in A549/IR cell lines, which further indicates the radioresistant phenotype.

Another interesting finding is that the increase in radiosensitivity in A549/IR cells may have been due to the inhibition ability of DIO on Akt activation. As previously mentioned, a critical event that determines the cancer radioresistance is the aberrantly activated Akt signaling [33]. Suppression of Akt signaling could enhance the efficacy of radiation therapy in human cancer cells [34]. From the western blot and cell proliferation rate assays, we found that DIO with or without RT could inhibit the Akt Ser473 phosphorylation level. In the meanwhile, down-regulation of the Akt signaling pathway by DIO or inhibitors (LY294002 and MK-2206) results in enhanced radiosensitivity in A549/IR cell lines. As used Akt inhibitors (LY294002 and MK-2206) as the positive control, we conclude that DIO could serve as a potential inhibitor for Akt signaling pathway. Through relieving the Akt activity, DIO could effectively down-regulate the downstream signal of Akt in the radioresistant A549/IR cell lines. Compared with the parent cells A549, the cytotoxicity of ALO on A549/IR are more remarkable and selective, further indicating the radio-resistance phenotype of A549/IR cells. Meanwhile, the basal Akt activation level, marked by the Akt ser473, is much higher in A549/IR cells. And DIO could preferentially inhibit the higher Akt activation level, similar to the Akt inhibitor LY294002 and MK-2206. Because of lower basal Akt activation, the parent cells A549 shows relative resistance to DIO, serving as an Akt inhibitor. Taken together, DIO is only used to treat the A549/IR cells with high Akt activation.

In addition, growing evidence indicates that, apart from the cell cycle changes and DNA damage response, other elements are also proved to be associated with the radioresistance of lung cancer cells, including increased epithelial-mesenchymal transition (EMT) phenotypes and elevated growth factors [35, 36]. Therefore, a better understanding of the potential molecular mechanism underlying the radiosensitizing effect of DIO is of great importance for lung cancer research in the further.

In conclusion, we demonstrated that agent DIO enhancement of the radiosensitivity of lung cancer cells is dependent on the Akt signaling pathway. Our studies suggest that DIO could effectively improve the therapeutic effects of RT.

## Supporting information

S1 Fig Effects of DIO on the cell radiosensitivity in parental A549 cells. (A) A549 cell lines were treated with indicated conditions for 24, 48, and 72 h. Cell viability was evaluated using the MTS assay. (B) Cell cycle-related proteins (CDK4/6, CyclinD1 and CDC2) in A549 cells were determined using western blot analysis. β-actin was used for the loading control.
(TIF)

S2 Fig DIO on the DNA damage makers in A549/IR cells. A549/IR cell lines were treated with indicated conditions. Cell lysates were processed for the indicated proteins by immunoblotting. β-actin expression shows the equal loading.
(TIF)

## References

[1] Eberhardt WE, Stuschke M. Multimodal treatment of non-small-cell lung cancer. Lancet. 2015;386(9998):1018–20. doi: 10.1016/S0140-6736(15)61083-2 26275734 10.1016/S0140-6736(15)61083-2

[2] Guo W, Xie L, Zhao L, Zhao Y. mRNA and microRNA expression profiles of radioresistant NCI-H520 non-small cell lung cancer cells. Molecular medicine reports. 2015;12(2):1857–67. PubMed Central PMCID: PMC4464398. doi: 10.3892/mmr.2015.3600 25873351 10.3892/mmr.2015.3600 PMC4464398

[3] Choi SH, Yang H, Lee SH, Ki JH, Nam DH, Yoo HY. TopBP1 and Claspin contribute to the radioresistance of lung cancer brain metastases. Molecular cancer. 2014;13:211 PubMed Central PMCID: PMC4168047. doi: 10.1186/1476-4598-13-211 25216549 10.1186/1476-4598-13-211 PMC4168047

[4] Mahmood J, Zaveri SR, Murti SC, Alexander AA, Connors CQ, Shukla HD, et al Caveolin-1: a novel prognostic biomarker of radioresistance in cancer. International journal of radiation biology. 2016;92(12):747–53. doi: 10.1080/09553002.2016.1222096 27623870 10.1080/09553002.2016.1222096

[5] Mundi PS, Sachdev J, McCourt C, Kalinsky K. AKT in cancer: new molecular insights and advances in drug development. British journal of clinical pharmacology. 2016;82(4):943–56. doi: 10.1111/bcp.13021 27232857 10.1111/bcp.13021 PMC5137819

[6] Mayer IA, Arteaga CL. The PI3K/AKT Pathway as a Target for Cancer Treatment. Annual review of medicine. 2016;67:11–28. doi: 10.1146/annurev-med-062913-051343 26473415 10.1146/annurev-med-062913-051343

[7] Karagounis IV, Kalamida D, Mitrakas A, Pouliliou S, Liousia MV, Giatromanolaki A, et al Repression of the autophagic response sensitises lung cancer cells to radiation and chemotherapy. British journal of cancer. 2016;115(3):312–21. PubMed Central PMCID: PMC4973160. doi: 10.1038/bjc.2016.202 27380135 10.1038/bjc.2016.202 PMC4973160

[8] Zhang L, Huang Y, Zhuo W, Zhu Y, Zhu B, Chen Z. Fisetin, a dietary phytochemical, overcomes Erlotinib-resistance of lung adenocarcinoma cells through inhibition of MAPK and AKT pathways. American journal of translational research. 2016;8(11):4857–68. PubMed Central PMCID: PMC5126328. 27904686 PMC5126328

[9] Yim JH, Yun HS, Lee SJ, Baek JH, Lee CW, Song JY, et al Radiosensitizing effect of PSMC5, a 19S proteasome ATPase, in H460 lung cancer cells. Biochemical and biophysical research communications. 2016;469(1):94–100. doi: 10.1016/j.bbrc.2015.11.077 26592665 10.1016/j.bbrc.2015.11.077

[10] Ochodnicka-Mackovicova K, Bahjat M, Bloedjes TA, Maas C, de Bruin AM, Bende RJ, et al NF-kappaB and AKT signaling prevent DNA damage in transformed pre-B cells by suppressing RAG1/2 expression and activity. Blood. 2015;126(11):1324–35. PubMed Central PMCID: PMC4671331. doi: 10.1182/blood-2015-01-621623 26153519 10.1182/blood-2015-01-621623 PMC4671331

[11] Gao H, Xue J, Zhou L, Lan J, He J, Na F, et al Bevacizumab radiosensitizes non-small cell lung cancer xenografts by inhibiting DNA double-strand break repair in endothelial cells. Cancer letters. 2015;365(1):79–88. doi: 10.1016/j.canlet.2015.05.011 25982206 10.1016/j.canlet.2015.05.011

[12] Marampon F, Gravina G, Ju X, Vetuschi A, Sferra R, Casimiro M, et al Cyclin D1 silencing suppresses tumorigenicity, impairs DNA double strand break repair and thus radiosensitizes androgen-independent prostate cancer cells to DNA damage. Oncotarget. 2016;7(5):5383–400. PubMed Central PMCID: PMC4868693. doi: 10.18632/oncotarget.6579 26689991 10.18632/oncotarget.6579 PMC4868693

[13] Androutsopoulos V, Wilsher N, Arroo RR, Potter GA. Bioactivation of the phytoestrogen diosmetin by CYP1 cytochromes P450. Cancer letters. 2009;274(1):54–60. doi: 10.1016/j.canlet.2008.08.032 18976853 10.1016/j.canlet.2008.08.032

[14] Zhan G, Pan L, Tu K, Jiao S. Antitumor, Antioxidant, and Nitrite Scavenging Effects of Chinese Water Chestnut (Eleocharis dulcis) Peel Flavonoids. Journal of food science. 2016;81(10):H2578–H86. doi: 10.1111/1750-3841.13434 27603811 10.1111/1750-3841.13434

[15] You S, Li R, Park D, Xie M, Sica GL, Cao Y, et al Disruption of STAT3 by niclosamide reverses radioresistance of human lung cancer. Molecular cancer therapeutics. 2014;13(3):606–16. PubMed Central PMCID: PMC3964811. doi: 10.1158/1535-7163.MCT-13-0608 24362463 10.1158/1535-7163.MCT-13-0608 PMC3964811

[16] Lu J, Tang M, Li H, Xu Z, Weng X, Li J, et al EBV-LMP1 suppresses the DNA damage response through DNA-PK/AMPK signaling to promote radioresistance in nasopharyngeal carcinoma. Cancer letters. 2016;380(1):191–200. doi: 10.1016/j.canlet.2016.05.032 27255972 10.1016/j.canlet.2016.05.032

[17] Yang L, Xu Z, Liu L, Luo X, Lu J, Sun L, et al Targeting EBV-LMP1 DNAzyme enhances radiosensitivity of nasopharyngeal carcinoma cells by inhibiting telomerase activity. Cancer biology & therapy. 2014;15(1):61–8. PubMed Central PMCID: PMC3938524. 24145206 10.4161/cbt.26606 PMC3938524

[18] Ma X, Xu Z, Yang L, Xiao L, Tang M, Lu J, et al EBV-LMP1-targeted DNAzyme induces DNA damage and causes cell cycle arrest in LMP1-positive nasopharyngeal carcinoma cells. International journal of oncology. 2013;43(5):1541–8. doi: 10.3892/ijo.2013.2098 24042231 10.3892/ijo.2013.2098

[19] Zeng XJ, Shi J, Zhao M, Chen QW, Wang LP, Jiang HY, et al Regioselective Glucuronidation of Diosmetin and Chrysoeriol by the Interplay of Glucuronidation and Transport in UGT1A9-Overexpressing HeLa Cells. Plos One. 2016;11(11):e0166239 doi: 10.1371/journal.pone.0166239 27832172 10.1371/journal.pone.0166239 PMC5104480

[20] Liu P, Begley M, Michowski W, Inuzuka H, Ginzberg M, Gao D, et al Cell-cycle-regulated activation of Akt kinase by phosphorylation at its carboxyl terminus. Nature. 2014;508(7497):541–5. PubMed Central PMCID: PMC4076493. doi: 10.1038/nature13079 24670654 10.1038/nature13079 PMC4076493

[21] Visconti R, Della Monica R, Grieco D. Cell cycle checkpoint in cancer: a therapeutically targetable double-edged sword. Journal of experimental & clinical cancer research: CR. 2016;35(1):153. PubMed Central PMCID: PMC5037895. 27670139 10.1186/s13046-016-0433-9 PMC5037895

[22] Ruijtenberg S, van den Heuvel S. G1/S Inhibitors and the SWI/SNF Complex Control Cell-Cycle Exit during Muscle Differentiation. Cell. 2015;162(2):300–13. doi: 10.1016/j.cell.2015.06.013 26144318 10.1016/j.cell.2015.06.013

[23] Wada T, Joza N, Cheng HY, Sasaki T, Kozieradzki I, Bachmaier K, et al MKK7 couples stress signalling to G2/M cell-cycle progression and cellular senescence. Nature cell biology. 2004;6(3):215–26. doi: 10.1038/ncb1098 15039780 10.1038/ncb1098

[24] Morgan MA, Lawrence TS. Molecular Pathways: Overcoming Radiation Resistance by Targeting DNA Damage Response Pathways. Clinical cancer research: an official journal of the American Association for Cancer Research. 2015;21(13):2898–904. PubMed Central PMCID: PMC4494107. 26133775 10.1158/1078-0432.CCR-13-3229 PMC4494107

[25] Stover EH, Konstantinopoulos PA, Matulonis UA, Swisher EM. Biomarkers of Response and Resistance to DNA Repair Targeted Therapies. Clinical cancer research: an official journal of the American Association for Cancer Research. 2016;22(23):5651–60. 27678458 10.1158/1078-0432.CCR-16-0247

[26] Lorat Y, Schanz S, Rube CE. Ultrastructural Insights into the Biological Significance of Persisting DNA Damage Foci after Low Doses of Ionizing Radiation. Clinical cancer research: an official journal of the American Association for Cancer Research. 2016;22(21):5300–11. 27199493 10.1158/1078-0432.CCR-15-3081

[27] Chang L, Graham PH, Hao J, Ni J, Bucci J, Cozzi PJ, et al PI3K/Akt/mTOR pathway inhibitors enhance radiosensitivity in radioresistant prostate cancer cells through inducing apoptosis, reducing autophagy, suppressing NHEJ and HR repair pathways. Cell death & disease. 2014;5:e1437. PubMed Central PMCID: PMC4237243. 25275598 10.1038/cddis.2014.415 PMC4237243

[28] Chen JC, Hsieh MJ, Chen CJ, Lin JT, Lo YS, Chuang YC, et al Polyphyllin G induce apoptosis and autophagy in human nasopharyngeal cancer cells by modulation of AKT and mitogen-activated protein kinase pathways in vitro and in vivo. Oncotarget. 2016;7(43):70276–70289. doi: 10.18632/oncotarget.11839 27602962 10.18632/oncotarget.11839 PMC5342552

[29] Wisinski KB, Tevaarwerk AJ, Burkard ME, Rampurwala M, Eickhoff J, Bell MC, et al Phase I Study of an AKT Inhibitor (MK-2206) Combined with Lapatinib in Adult Solid Tumors Followed by Dose Expansion in Advanced HER2+ Breast Cancer. Clinical cancer research: an official journal of the American Association for Cancer Research. 2016;22(11):2659–67. PubMed Central PMCID: PMC4891227. 27026198 10.1158/1078-0432.CCR-15-2365 PMC4891227

[30] Cheng G, Zielonka J, Ouari O, Lopez M, McAllister D, Boyle K, et al Mitochondria-Targeted Analogues of Metformin Exhibit Enhanced Antiproliferative and Radiosensitizing Effects in Pancreatic Cancer Cells. Cancer research. 2016;76(13):3904–15. PubMed Central PMCID: PMC4930686. doi: 10.1158/0008-5472.CAN-15-2534 27216187 10.1158/0008-5472.CAN-15-2534 PMC4930686

[31] Kriegs M, Gurtner K, Can Y, Brammer I, Rieckmann T, Oertel R, et al Radiosensitization of NSCLC cells by EGFR inhibition is the result of an enhanced p53-dependent G1 arrest. Radiotherapy and oncology: journal of the European Society for Therapeutic Radiology and Oncology. 2015;115(1):120–7. 25796091 10.1016/j.radonc.2015.02.018

[32] Bhattacharjee S, Nandi S. Choices have consequences: the nexus between DNA repair pathways and genomic instability in cancer. Clinical and translational medicine. 2016;5(1):45 doi: 10.1186/s40169-016-0128-z 27921283 10.1186/s40169-016-0128-z PMC5136664

[33] Bussink J, van der Kogel AJ, Kaanders JH. Activation of the PI3-K/AKT pathway and implications for radioresistance mechanisms in head and neck cancer. The Lancet Oncology. 2008;9(3):288–96. doi: 10.1016/S1470-2045(08)70073-1 18308254 10.1016/S1470-2045(08)70073-1

[34] Dadey DY, Kapoor V, Hoye K, Khudanyan A, Collins A, Thotala D, et al Antibody targeting GRP78 enhances the efficacy of radiation therapy in human glioblastoma and non-small-cell lung cancer cell lines and tumor models. Clinical cancer research: an official journal of the American Association for Cancer Research. 2016. 10.1158/1078-0432.CCR-16-1935 27815359

[35] Gomez-Casal R, Bhattacharya C, Ganesh N, Bailey L, Basse P, Gibson M, et al Non-small cell lung cancer cells survived ionizing radiation treatment display cancer stem cell and epithelial-mesenchymal transition phenotypes. Molecular cancer. 2013;12(1):94 PubMed Central PMCID: PMC3751356. doi: 10.1186/1476-4598-12-94 23947765 10.1186/1476-4598-12-94 PMC3751356

[36] Gomez-Casal R, Epperly MW, Wang H, Proia DA, Greenberger JS, Levina V. Radioresistant human lung adenocarcinoma cells that survived multiple fractions of ionizing radiation are sensitive to HSP90 inhibition. Oncotarget. 2015;6(42):44306–22. PubMed Central PMCID: PMC4792558. doi: 10.18632/oncotarget.6248 26517240 10.18632/oncotarget.6248 PMC4792558

