# Supplementary figures and images for: Radiosensitizing effect of diosmetin on radioresistant lung cancer cells via Akt signaling pathway
Source: PLoS One. 2017 Apr 17;12(4):e0175977. doi: 10.1371/journal.pone.0175977 (PMC5393875; doi:10.1371/journal.pone.0175977)

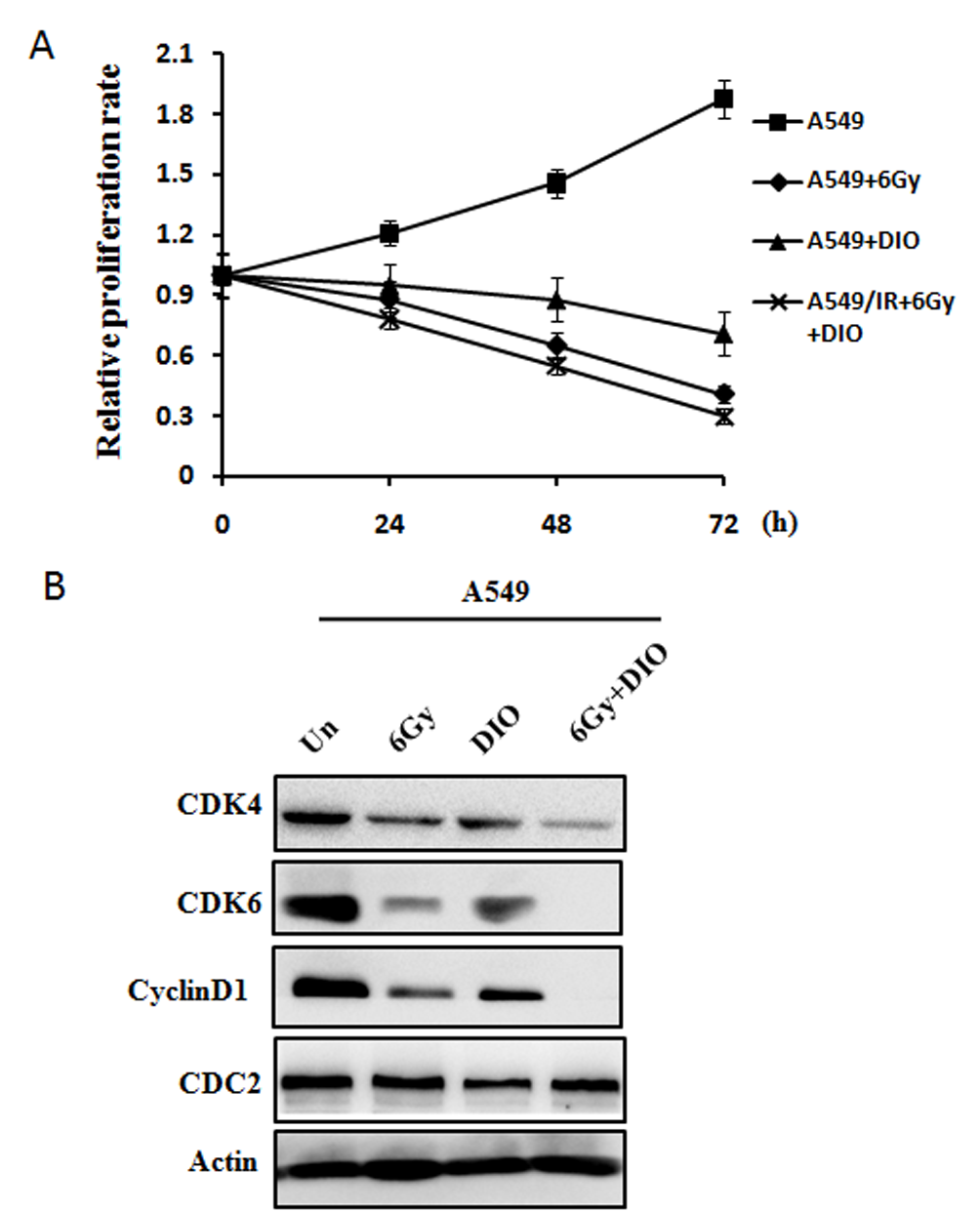

Supplement: S1 Fig — (A) A549 cell lines were treated with indicated conditions for 24, 48, and 72 h. Cell viability was evaluated using the MTS assay. (B) Cell cycle-related proteins (CDK4/6, CyclinD1 and CDC2) in A549 cells were determined using western blot analysis. β-actin was used for the loading control. (TIF) [file pone.0175977.s001.tif]

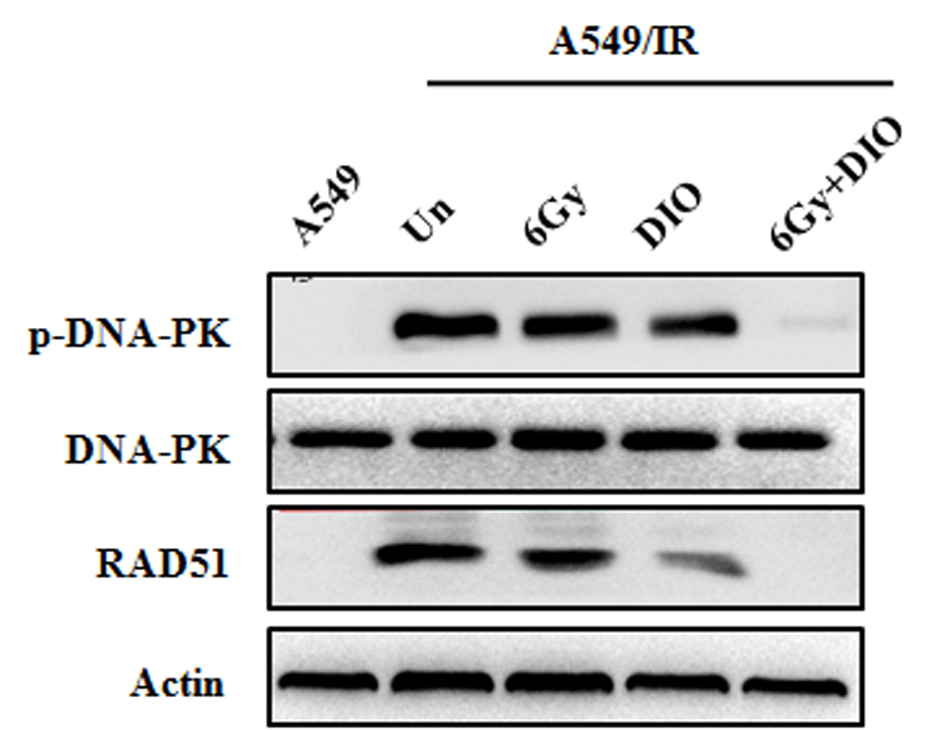

Supplement: S2 Fig — A549/IR cell lines were treated with indicated conditions. Cell lysates were processed for the indicated proteins by immunoblotting. β-actin expression shows the equal loading. (TIF) [file pone.0175977.s002.tif]
